# Deep Learning and Machine Learning Algorithms for Cervical Cancer Segmentation on MRI: A Systematic Review

**DOI:** 10.1002/jmrs.70127

**Published:** 2026-09-27

**Authors:** Somayeh Haji Ahmadi, Sina Soltan Mohammadi, Abolfazl Koozari, Mohammadreza Elhaie

**Affiliations:** ^1^ Department of Radiology, School of Medicine Isfahan University of Medical Sciences Isfahan Iran; ^2^ Department of Medical Physics, School of Medicine Ahvaz Jundishapur University of Medical Sciences Ahvaz Iran

**Keywords:** deep learning, image segmentation, machine learning, magnetic resonance imaging, uterine cervical neoplasms

## Abstract

**Introduction:**

Cervical cancer management relies heavily on magnetic resonance imaging (MRI) for staging and treatment planning; however, manual segmentation is time‐consuming and prone to variability. This systematic review aimed to evaluate the performance and clinical potential of machine learning (ML) and deep learning (DL) algorithms for automated cervical cancer segmentation on MRI.

**Methods:**

A systematic review guided by the Preferred Reporting Items for Systematic Reviews and Meta‐Analyses (PRISMA) (PROSPERO: CRD420251247441) was conducted by searching PubMed/MEDLINE, Embase, Scopus, Web of Science, IEEE Xplore, and Google Scholar until December 2025. Two reviewers independently screened studies, extracted data, and assessed risk of bias using the Quality Assessment of Diagnostic Accuracy Studies‐2 (QUADAS‐2). MRI‐based segmentation studies were primarily included, while computed tomography (CT)‐ and positron emission tomography (PET)‐based multimodal studies were analysed as complementary evidence. Due to substantial heterogeneity, findings were synthesized narratively, and certainty of evidence was assessed using the Grading of Recommendations Assessment, Development and Evaluation (GRADE) framework.

**Results:**

Thirty studies were included, mainly evaluating segmentation of tumours, high‐risk clinical target volumes, and organs at risk. DL models, particularly U‐Net variants, convolutional neural networks, and transformer‐based architectures, demonstrated promising performance, with reported Dice similarity coefficients ranging from 0.60 to 0.93. However, performance varied according to target structure, tumour characteristics, and imaging protocols. Overall risk of bias showed “some concerns,” mainly due to patient selection, reference standards, and limited external validation. GRADE assessment indicated low certainty of evidence due to heterogeneity, methodological limitations, and imprecision.

**Conclusion:**

ML and DL algorithms show promising potential for automated cervical cancer segmentation on MRI, with potential benefits for efficiency and reproducibility. However, further multicenter studies with standardized protocols and external validation are required before routine clinical implementation.

**Trial Registration:**

PRISMA guidelines and was prospectively registered in PROSPERO: CRD420251247441

## Graphical abstract

This systematic review summarizes current evidence on machine and deep learning algorithms for MRI‐based cervical cancer segmentation, demonstrating promising diagnostic performance and potential for workflow automation. However, methodological heterogeneity, limited external validation, and low certainty of evidence highlight the need for standardized multicenter studies before clinical implementation.

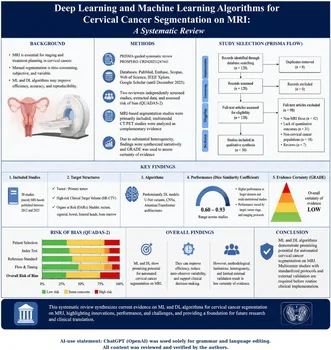

## 1 Introduction

Cervical cancer ranks as the fourth most prevalent malignancy among women globally, with the World Health Organization reporting approximately 660,000 new cases and 350,000 deaths in 2022, predominantly in low‐ and middle‐income countries where screening and treatment access remains limited [1, 2]. Magnetic resonance imaging (MRI) serves as a vital tool in cervical cancer management, providing superior soft‐tissue contrast for accurate tumour staging, assessment of local invasion, and evaluation of lymph node involvement, which inform treatment decisions [3]. Advanced MRI sequences, such as diffusion‐weighted imaging (DWI) and dynamic contrast‐enhanced (DCE) imaging, offer additional insights into tumour biology, acting as non‐invasive prognostic biomarkers [4, 5]. Image segmentation (delineating tumour boundaries and adjacent structures) is crucial for radiotherapy planning, surgical guidance, and volumetric analysis, yet manual methods are labor‐intensive, subjective, and variable across observers [6, 7]. Machine learning (ML) and deep learning (DL) algorithms, including convolutional neural networks (CNNs) and U‐Net variants, have transformed automated segmentation by effectively managing MRI complexities and enhancing accuracy [8, 9, 10]. Although reviews exist on DL for cervical cancer detection via cytology or colposcopy, and artificial intelligence (AI) in gynaecological cancers broadly, a targeted synthesis on ML/DL for MRI‐based cervical cancer segmentation is lacking. This systematic review, following the Preferred Reporting Items for Systematic Reviews and Meta‐Analyses (PRISMA) guidelines, synthesizes evidence on ML and DL algorithms for cervical cancer segmentation on MRI, evaluating performance, innovations, and clinical utility in studies up to December 2025. It identifies challenges in generalizability, data availability, and workflow integration while proposing future directions for AI in cervical oncology. Although MRI is a major imaging modality for cervical cancer segmentation because of its high soft‐tissue contrast, recent AI workflows increasingly integrate complementary imaging modalities such as computed tomography (CT) and positron emission tomography (PET). Therefore, multimodal studies were considered to capture emerging AI‐assisted segmentation strategies while maintaining MRI‐based segmentation as the central focus of this review.

## 2 Methods

This systematic review adhered to the Preferred Reporting Items for Systematic Reviews and Meta‐Analyses (PRISMA) guidelines and was prospectively registered with PROSPERO (CRD420251247441) [11, 12].

### 2.1 Eligibility Criteria

Studies were eligible if they evaluated ML or DL‐based automated segmentation approaches in cervical cancer imaging. The primary focus was MRI‐based segmentation; however, studies incorporating multimodal approaches, including PET/MRI, CT/MRI fusion, or CT‐assisted segmentation, were also included when MRI‐derived information was an essential component of the segmentation workflow or when the investigated algorithm was directly transferable to MRI‐guided clinical applications. These studies were analysed separately as multimodal extensions rather than as pure MRI segmentation studies. Therefore, multimodal and CT‐assisted studies were not pooled with MRI‐only studies but were discussed separately to avoid overestimating MRI‐specific segmentation performance. This represented a post‐protocol clarification because several eligible studies incorporated MRI‐derived information within multimodal radiotherapy workflows. These studies were retained to reflect emerging AI‐assisted multimodal segmentation strategies but were not considered equivalent to MRI‐only evidence.

### 2.2 Search Strategy

A comprehensive literature search was conducted without date or language restrictions to identify relevant studies. The following databases were searched: PubMed/MEDLINE, Embase, Scopus, Web of Science, IEEE Xplore, and Google Scholar. Search terms were structured around the Population, Intervention, Comparator, and Outcome (PICO) framework, combined using Boolean operators (AND, OR, NOT). Table S4 outlines the PICO elements and corresponding search strategy.

Database‐specific adaptations were applied. For PubMed/MEDLINE: (MRI OR “magnetic resonance imaging”) AND (segmentation OR “image segmentation” OR “tumour segmentation” OR “automatic segmentation”) AND (“cervical cancer” OR “cervix cancer” OR “cervical carcinoma” OR “cervical neoplasms”[MeSH]) AND (“diagnostic performance” OR “diagnostic accuracy” OR sensitivity OR specificity OR AUC OR ROC OR “predictive value”) NOT (review[pt] OR “systematic review”[tiab] OR “meta‐analysis”[pt] OR meta‐analysis[tiab]). Similar tailored strategies were used for Embase, Scopus, Web of Science, and IEEE Xplore (full strategies available in the PROSPERO protocol).

### 2.3 Study Selection

Titles and abstracts were screened independently by two reviewers using predefined eligibility criteria. Full‐text articles were retrieved for potentially eligible studies, with discrepancies resolved through discussion or consultation with a third reviewer if needed. Only published studies were considered; unpublished or grey literature was excluded.

### 2.4 Data Extraction

Data were extracted independently by two reviewers using a standardized form. Extracted items included study characteristics, algorithm details, performance metrics, and secondary outcomes. Authors were not contacted for missing data.

### 2.5 Quality Assessment

Risk of bias and applicability concerns were assessed independently by two reviewers using the Quality Assessment of Diagnostic Accuracy Studies‐2 (QUADAS‐2) tool [13]. The study‐level risk of bias summary and applicability assessments are provided in Tables S1 and S2, respectively, while detailed domain‐specific justifications are presented in Table S3.

### 2.6 Data Synthesis

If sufficient homogeneity in outcomes and metrics was present, a random‐effects meta‐analysis was planned to pool performance measures. Otherwise, a narrative synthesis was conducted, summarizing algorithm types, methodological characteristics, limitations, and clinical implications. Subgroup analyses explored influences of MRI sequences and architectures.

### 2.7 Certainty of Evidence

Although not predefined in the protocol, the GRADE approach was applied post hoc to assess the certainty of evidence for major outcomes. This exploratory assessment considered risk of bias, inconsistency, indirectness, imprecision, and publication bias domains.

## 3 Results

### 3.1 Study Selection

The literature search yielded 1256 unique records after duplicate removal. Following title and abstract screening, 128 full‐text articles were assessed for eligibility. Full‐text assessment resulted in exclusion of 98 studies. The main exclusion reasons were non‐relevant imaging modality (*n* = 42), absence of quantitative segmentation outcomes (*n* = 31), non‐cervical cancer populations (*n* = 18), and review or meta‐analysis articles (*n* = 7), as summarized in Table S5. Ultimately, 30 studies met the inclusion criteria and were incorporated into the synthesis. The study selection process is summarized in the PRISMA flow diagram shown in Figure 1.

**FIGURE 1:**
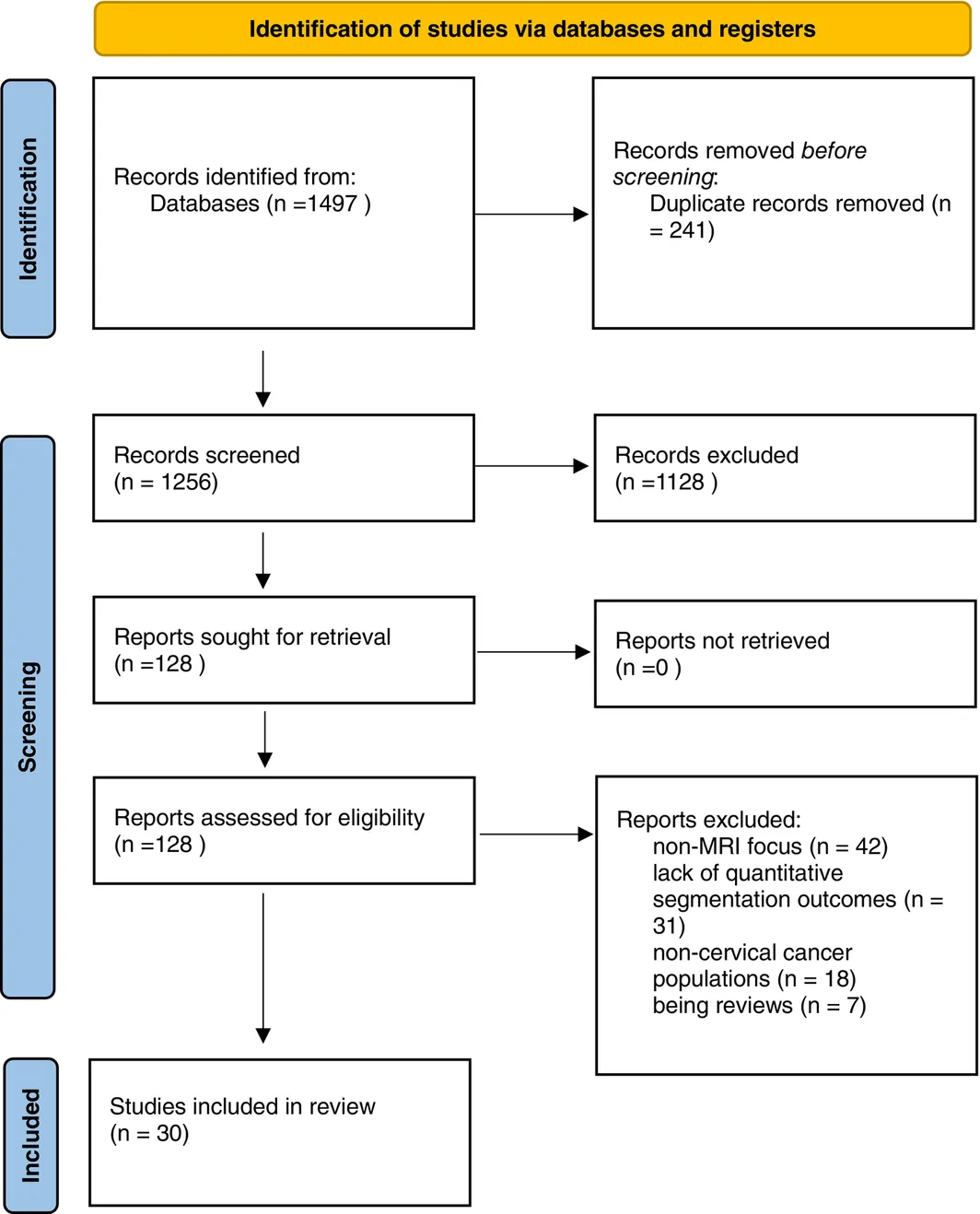
PRISMA flow diagram of the study selection process.

### 3.2 Risk of Bias Assessment

The methodological quality of the included studies was evaluated using the QUADAS‐2 framework across four domains: patient selection, index test, reference standard, and flow and timing (Figures 2 and 3). The results of this assessment are summarized in the traffic‐light plot and the overall distribution figure. Overall, the risk of bias was predominantly judged as “some concerns,” indicating moderate methodological limitations across the evidence base. Study‐level QUADAS‐2 assessments demonstrated variability across the included studies (Table S1). Concerns were most frequently related to patient selection, reference standard definition, and limited external validation of AI models. Applicability assessments were generally favourable, although several studies showed concerns related to restricted datasets, non‐representative populations, and limited generalizability (Table S2). A smaller proportion of studies were rated as low risk of bias, whereas a non‐negligible subset was classified as high risk, contributing to reduced confidence in certain findings. Across domains, flow and timing demonstrated the most favourable profile, with the vast majority of studies rated as low risk, suggesting that patient progression through the study, timing between index and reference tests, and completeness of outcome verification were generally appropriate. In contrast, patient selection showed the greatest vulnerability, with a notable proportion of studies exhibiting high risk of bias, likely reflecting non‐consecutive enrollment, case–control designs, or insufficient reporting of inclusion and exclusion criteria. The index test domain was largely assessed as low risk or some concerns, implying that although MRI‐based assessments were generally implemented appropriately, blinding to reference standards and prespecification of diagnostic thresholds were not consistently reported. Similarly, the reference standard domain frequently raised some concerns, suggesting potential uncertainty regarding whether reference diagnoses were independent, uniformly applied, or sufficiently rigorous across studies. Taken together, these findings indicate that while several studies demonstrated robust methodological quality, the overall evidence is limited by variability in study design and reporting—particularly in patient selection and reference standard procedures—which should be considered when interpreting pooled estimates and conclusions.

**FIGURE 2:**
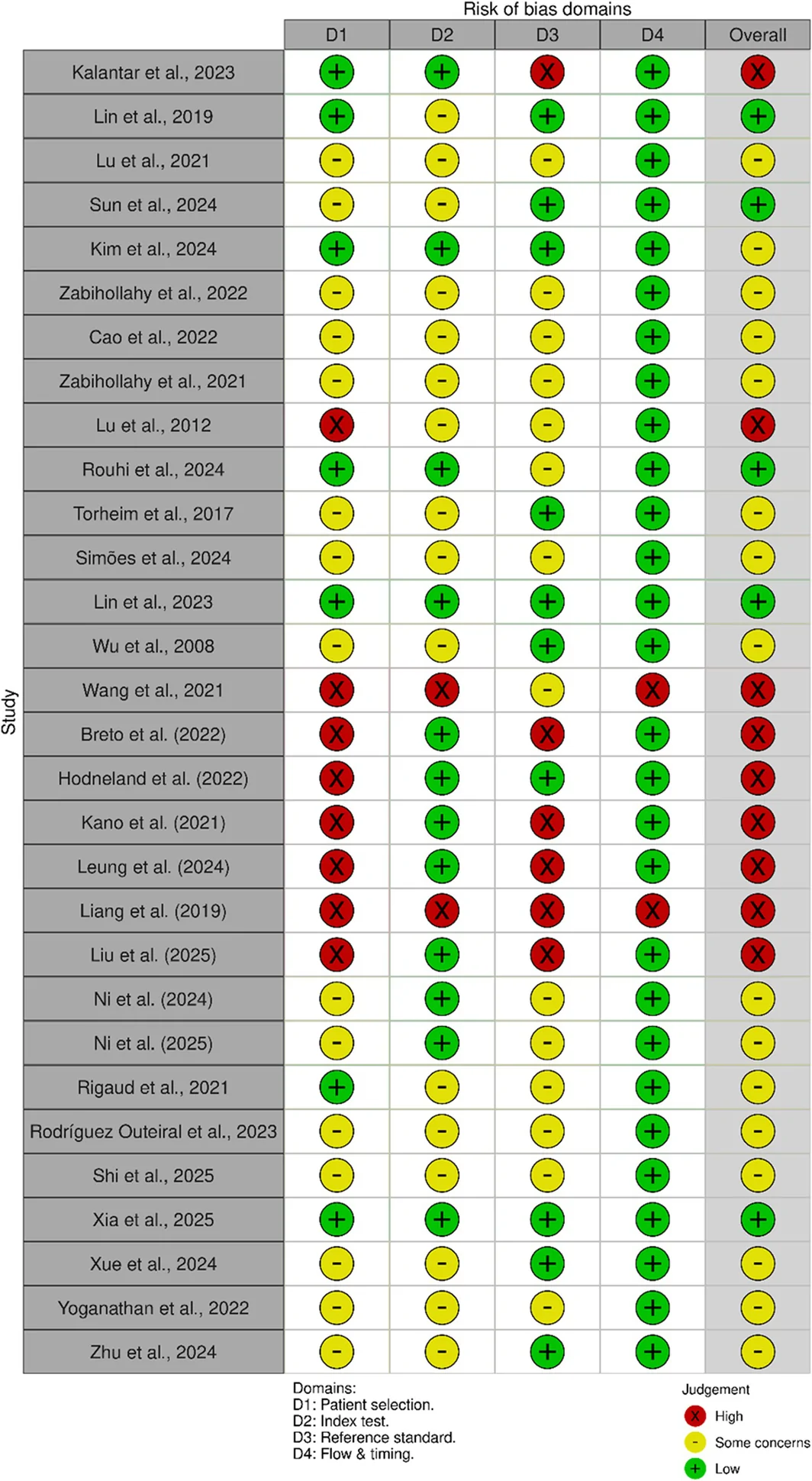
Risk‐of‐bias assessment results across 30 included studies in four domains (patient selection, index test, reference standard, and flow and timing).

**FIGURE 3:**
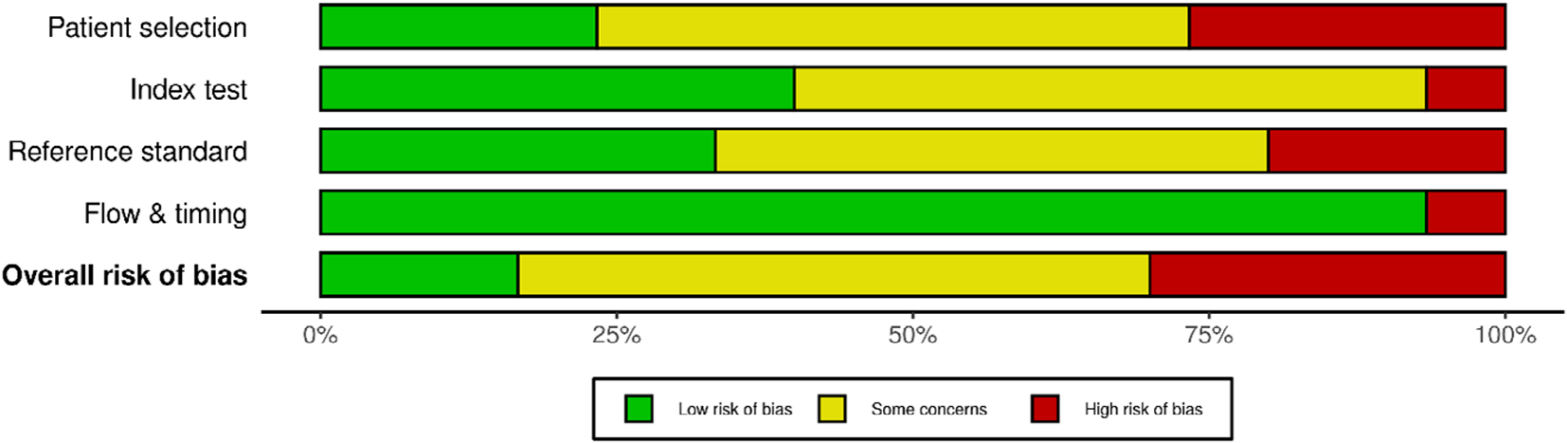
Risk‐of‐bias assessment summary of the 30 included studies using the QUADAS‐2 tool.

### 3.3 The Certainty of Evidence

The certainty of evidence was evaluated using the GRADE framework and was rated as high, moderate, low, or very low according to the presence of limitations in risk of bias, inconsistency, indirectness, imprecision, and publication bias. Overall, the certainty of evidence for AI‐based cervical cancer segmentation outcomes was judged to be low, primarily due to methodological limitations, substantial heterogeneity in imaging protocols, segmentation targets, algorithm architectures, and performance metrics, as well as limited external validation and relatively small sample sizes in several studies. Although many studies reported promising segmentation performance, including high Dice similarity coefficient values for selected targets, the variability in study design and reporting reduced confidence in the generalizability of these findings. No outcome was considered to provide high‐certainty evidence, and the available evidence should therefore be interpreted as preliminary until validated through larger, multicenter, and externally validated studies (Table 1).

**TABLE 1:** GRADE summary of findings.

Outcome	Number of studies (patients)	Summary of findings	Risk of bias	Inconsistency	Indirectness	Imprecision	Publication bias	Overall certainty of evidence
DSC for GTV/HR‐CTV Segmentation	25 studies (approx. 3500 patients across studies)	Median DSC ranged from 0.60 to 0.93 (higher for larger/homogeneous tumours; lower for small/regressing tumours). DL models, including U‐Net variants and transformer‐based architectures, generally demonstrated promising segmentation performance; however, direct comparisons with traditional methods were limited and heterogeneous	Serious (−1): QUADAS‐2 showed “some concerns” overall, with high risk in patient selection (e.g., non‐consecutive enrollment, single‐center bias) and reference standards (e.g., variable expert delineations)	Serious (−1): High heterogeneity in DSC due to differences in MRI sequences (e.g., T2W vs. multiparametric), tumour stages, and model architectures; no meta‐analysis possible	Not serious: Studies directly addressed cervical cancer populations and MRI‐based segmentation relevant to clinical staging/radiotherapy	Serious (−1): Wide ranges in reported DSC (e.g., 0.44–0.93) with small sample sizes in some studies (< 50 patients), leading to imprecise estimates	Undetected: Not formally assessed, but potential for positive results bias as negative/unfavourable outcomes may be underreported	Low (downgraded by 3 levels from High)
DSC for OAR Segmentation	15 studies (approx. 2000 patients across studies)	Mean/median DSC ranged from 0.66 to 0.93 (higher for rigid/well‐defined OARs like bladder; lower for deformable structures like sigmoid/rectum)	Serious (−1): Similar QUADAS‐2 concerns as above, with additional issues in flow/timing for multi‐organ tasks	Serious (−1): Variability across OAR types and imaging protocols; e.g., DSC 0.93 for bladder but 0.66 for sigmoid in some studies	Not serious: Directly applicable to radiotherapy planning in cervical cancer	Serious (−1): Broad confidence intervals implied by ranges; limited reporting of variability in smaller cohorts	Undetected: Possible, given focus on successful models	Low (downgraded by 3 levels from High)
Secondary Metrics (e.g., HD, Dosimetric Impacts)	20 studies (subset of above)	HD typically 3–6 mm; dosimetric differences minimal (e.g., ΔD90 ~0.2 Gy); failure detection/uncertainty rarely reported	Serious (−1): As above	Serious (−1): Inconsistent metrics and reporting (e.g., 95% HD vs. mean HD)	Not serious	Serious (−1): Sparse data on clinical endpoints like dosimetric errors	Undetected	Low (downgraded by 3 levels from High)

Abbreviations: DL, deep learning; DSC, Dice similarity coefficient; GTV, gross tumour volume; HD, Hausdorff distance; HR‐CTV, high‐risk clinical target volume; MRI, magnetic resonance imaging; OAR, organs at risk.

### 3.4 Qualitative Synthesis

The included studies collectively advance the application of DL for automatic segmentation in cervical cancer imaging, primarily focusing on MRI, while also including selected CT‐only and multimodal CT/PET studies (Table 2). Importantly, multimodal studies were interpreted as complementary evidence rather than direct substitutes for MRI‐only segmentation performance. These investigations address key clinical challenges such as manual contouring variability, time inefficiency, and the need for robust, generalizable models to support radiotherapy planning, brachytherapy, and tumour monitoring. Most studies evaluate segmentation performance using metrics like DSC, Hausdorff distance (HD), and surface DSC, with comparisons to expert manual delineations. Innovations span from multi‐head architectures and transfer learning to multimodal fusion and failure detection mechanisms, demonstrating a progression toward clinically viable, automated workflows (Table S6).

**TABLE 2:** Data characterization of included studies.

Study	Sample size	Imaging modality	Evidence category	Target structures	DL/ML method	Validation strategy	External validation (Yes/No)	Key performance metrics
Kalantar et al. (2023) [14]	207 patients	Multiparametric MRI (T2W, DWI, ADC)	MRI‐only	Locally advanced cervical cancer tumours	Multi‐head dilated deep learning framework	Internal model comparison (baseline U‐Net vs. multi‐head models)	No	Median DSC: 0.823 (vs. 0.788 for baseline U‐Net)
Lin et al. (2020) [15]	169 patients	DWI (b0, b1000, ADC)	MRI‐only	Cervical cancer tumours	Triple‐channel U‐Net	Internal validation within single‐center dataset	No	DSC: 0.82; Sensitivity: 0.89
Lu et al. (2022) [16]	213 patients	Cervical MRI	MRI‐only (primary cervical MRI; secondary CT dataset for generalization evaluation)	Small cervical tumours	AugMS‐Net (3D Res2Net with multiscale supervision)	Evaluation on cervical MRI dataset with additional testing on LiTS CT dataset	No	Improved DSC over baselines; ~20% parameter reduction
Kim et al. (2024) [17]	65 patients	Pelvic MRI	MRI‐only	OARs (bladder, rectum, sigmoid) and HR‐CTV	Dual convolution–transformer UNet (coarse‐to‐fine, size‐adaptive)	Internal retrospective validation	No	DSC: 0.93 (bladder), 0.79 (rectum), 0.66 (sigmoid), 0.74 (HR‐CTV)
Zabihollahy et al. (2022) [18]	283 patients	T2W MRI	MRI‐only	Clinical target volume (CTV)	Anatomy‐guided cascaded (bladder localization + attention CTV detection +3D U‐Net)	Internal validation with Monte Carlo dropout uncertainty estimation	No	Mean DSC: 0.85 ± 0.03; 95% HD: 3.70 ± 0.35 mm
Cao et al. (2022) [19]	6 patients	Postimplant CT + preimplant MRI	MRI + CT multimodal	HR‐CTV	Dual‐path asymmetric CNN	Internal validation of dual‐path CNN model	No	DSC: 0.76 ± 0.06; 95% HD: ~6 mm
Zabihollahy et al. (2021) [20]	166 patients	Pelvic T2W MRI	MRI‐only	Multi‐organ (bladder, rectum, sigmoid)	Coarse‐to‐fine (3D U‐Net localization +3D Dense U‐Net segmentation)	Internal retrospective validation	No	Mean DSC: ~0.93 (bladder), 0.87–0.88 (rectum), 0.80 (sigmoid)
Lu et al. (2012) [21]	78 patients	Serial pelvic MRI	MRI‐only	Cervical tumours (with deformation and regression)	Bayesian‐based (integrated segmentation, registration, tumour detection)	Algorithm development and validation on available MRI dataset	No	DSC: 0.78–0.82 across treatment weeks
Rouhi et al. (2024) [22]	166 patients (115 training; 51 prospective test)	T2W MRI	MRI‐only	Gross tumour volume (GTV)	Ensemble 2D SegResNet (with radiomics failure detection)	115‐patient retrospective cohort for model development; separate 51‐patient prospective cohort for testing	No	DSC: 0.72 ± 0.16; Surface DSC: 0.66 ± 0.17
Torheim et al. (2017) [23]	78 patients	Multiparametric MRI (T2W, T1W, DCE)	MRI‐only	Locally advanced cervical tumours	Voxel‐wise classification (Fisher's Linear Discriminant Analysis)	Internal retrospective methodological evaluation	No	DSC: up to 0.44; Sensitivity/Specificity: ~0.84–0.95; AUC: up to 0.87
Simões et al. (2024) [24]	489 patients	MRI‐guided brachytherapy	MRI‐only	Target structures for brachytherapy	DL‐based auto‐segmentation (population‐based vs. patient‐specific)	Clinical workflow evaluation on MRI‐guided brachytherapy data	No	Reduced contouring time; DSC comparable to inter‐observer variability
Lin et al. (2023) [25]	513 patients	DWI MRI	MRI‐only	Uterine malignancies (cervical + others)	Transfer learning on U‐Net variants	Multicenter retrospective validation	No	DSC: 0.81 (combined), 0.91 (cervical alone)
Sun et al. (2024) [26]	52 patients	CT (with generated MR)	CT + generated MR multimodal	Clinical target volume (CTV)	AGSC‐GAN (MR generation) + multimodal segmentation	AI model development and validation using CT and generated MR datasets	No	DSC: 0.947; PSNR: 16.72; SSIM: 0.67
Wu et al. (2008) [27]	70 patients	MRI (across radiation stages)	MRI‐only	Cervical tumours	Iterative active deformational segmentation	Longitudinal internal evaluation across radiation treatment stages	No	Mean Jaccard: 0.71 (preradiation)
Wang et al. (2021) [28]	Not specified	Multisequence MRI	MRI‐only	Whole tumour, core tumour, enhanced tumour	3D‐CNN	Internal retrospective validation	No	DSC: 0.78 (whole), 0.64 (core), 0.71 (enhanced); Accuracy: 93.11%
Breto et al. (2022) [29]	15 patients	Planning and daily MRI	MRI‐only	GTV and OARs (rectum, bladder, femur, etc.)	Mask R‐CNN	Internal feasibility evaluation	No	DSC: > 0.8 (larger OARs), 0.6–0.7 (GTV, uterus, etc.)
Hodneland et al. (2022) [30]	131 patients	Multiparametric pelvic MRI	MRI‐only	Primary cervical tumours (whole‐volume)	3D U‐Net	Internal train/validation/test split (105 train/validation, 26 test)	No	DSC: ~0.60 (vs. raters)
Kano et al. (2021) [31]	98 patients	DWI MRI	MRI‐only	Gross tumour volume (GTV)	Combined 2D and 3D U‐Net (with model averaging)	K‐fold cross‐validation and model averaging	No	Median DSC: 0.83; Median HD: 4.7 mm
Leung et al. (2024) [32]	23 patients (52 MRI volumes)	3D MRI	MRI‐only	Tumours and OARs	Masked‐Net/dilated Masked‐Net (with transfer learning and positional Dice loss)	5‐fold cross‐validation	No	Average DSC: 0.79; Reduced HD/MSD
Liang et al. (2019) [33]	136 patients	Cervical cancer MRI	MRI‐focused; modality integration unclear	Cervical cancer segmentation	Optimized DL with wireless sensor network	Internal feasibility evaluation	No	Accuracy: ~98%–99%
Liu et al. (2025) [34]	165 patients	Multi‐modality PET/MRI	PET/MRI multimodal	Cervical tumours	Parallel encoder U‐Net (PEU‐Net)	Internal retrospective evaluation comparing PET/MRI models	No	Higher DSC and lower HD than baselines (specific values not detailed)
Ni et al. (2024) [35]	214 patients	MR‐guided brachytherapy	MRI‐only	Organs at risk (OARs)	nnU‐Net with transfer learning	Transfer learning evaluation across scanners/applicators	No	Restored DSC to original levels with fine‐tuning
Ni et al. (2025) [36]	195 patients	T2W MRI	MRI‐only	OARs (near‐target regions)	3D U‐Net with distance‐penalized loss	Internal testing cohort evaluation	No	Improved wDSC; Reduced D2cm^3^ discrepancies
Rigaud et al. (2021) [37]	222 patients	CT	CT‐only	Targets and OARs	2D DeepLabV3+ and 3D U‐Net	Internal validation and independent external validation	Yes	Good DSC agreement; Dose plans met clinical criteria
Rodríguez Outeiral et al. (2023) [38]	195 patients (524 fractions)	T2W MRI (brachytherapy)	MRI‐only	Gross tumour volume (GTV)	nnU‐Net	Internal retrospective evaluation	No	Median DSC: 0.73; ΔD90/ΔD98: ~0.2 Gy
Shi et al. (2025) [39]	222 patients (4063 images)	Small‐field T2W MRI (multi‐directional)	MRI‐only	Cervical cancer lesions	TransUnet (CNN + Transformer)	Internal retrospective evaluation	No	Average DSC: 0.7628; Lower mean HD than U‐Net
Xia et al. (2025) [40]	125 patients (> 14,000 slices)	Multi‐parametric MRI (DWI, T2W, CE‐T1W)	MRI‐only	Cervical cancer lesions	Cer‐ConvN3Unet (two‐stage detection + segmentation)	Multicenter retrospective development and validation	No	Detection accuracy: ~93%; DSC: ~0.83 (DWI), 0.79 (T2W), 0.74 (CE‐T1W)
Xue et al. (2024) [41]	135 patients	CT and MRI (brachytherapy)	Multimodal MRI‐based (CT + MRI)	HRCTV and OARs	3D Prompt‐ResUNet	Internal training/validation/test split (103 train, 16 validation, 16 test)	No	Mean DSC: ~0.92 (HRCTV/bladder), 0.87 (rectum)
Yoganathan et al. (2022) [42]	39 patients	MRI (brachytherapy)	MRI‐only	Targets (GTV, HR‐CTV) and OARs	2.5D DL models (ResNet50/InceptionResNetv2)	Internal retrospective validation	No	Better DSC and dose agreement with 2.5D InceptionResNetv2
Zhu et al. (2024) [43]	406 patients	MRI (CE‐T1W, T2W)	MRI‐only	Cervical tumours	CNN–Transformer (CoTr)	Internal retrospective validation	No	DSC: ~0.83 (CE‐T1W), 0.82 (T2W); C‐index: 0.733 for survival

Abbreviations: CT, computed tomography; MRI, magnetic resonance imaging; *n*, number of studies.

### 3.5 MRI‐Only Segmentation Studies

The majority of included studies evaluated artificial intelligence–based segmentation approaches using MRI as the primary imaging modality. These studies focused mainly on automated delineation of cervical tumours, gross tumour volume (GTV), high‐risk clinical target volume (HR‐CTV), and organs at risk (OARs) for radiotherapy planning and brachytherapy applications. MRI‐only studies demonstrated promising segmentation performance; however, reported accuracy varied substantially depending on tumour characteristics, imaging protocols, segmentation targets, and model architecture.

For tumour segmentation, Kalantar et al. developed a multi‐head dilated deep learning framework for multiparametric MRI segmentation and achieved a median Dice similarity coefficient (DSC) of 0.823, outperforming baseline multi‐channel U‐Net models (DSC 0.788). The study highlighted the value of combining T2‐weighted imaging and apparent diffusion coefficient (ADC) information for improving tumour boundary delineation. Lin et al. applied a triple‐channel U‐Net using diffusion‐weighted MRI (b0, b1000, and ADC), achieving a DSC of 0.82, sensitivity of 0.89, and strong correlation between automated and manual tumour volumes (*r* = 0.94). Similarly, Lu et al. proposed AugMS‐Net, an augmented multiscale network based on 3D Res2Net modules and deep multiscale supervision, improving small tumour segmentation performance while reducing model parameters by approximately 20%.

Several studies investigated advanced architectures for challenging tumour segmentation scenarios. Kim et al. introduced a dual convolution‐transformer U‐Net for simultaneous segmentation of HR‐CTV and OARs in MRI‐guided brachytherapy, achieving DSC values of 0.93 for bladder, 0.79 for rectum, 0.66 for sigmoid colon, and 0.74 for HR‐CTV. Zabihollahy et al. developed an anatomy‐guided cascaded CNN framework with Monte Carlo dropout‐based uncertainty estimation for CTV segmentation, achieving a DSC of 0.85 ± 0.03 and 95% Hausdorff distance (HD95) of 3.70 ± 0.35 mm. Another study by Zabihollahy et al. evaluated coarse‐to‐fine CNN models for multi‐organ pelvic MRI segmentation, reporting high performance for bladder (DSC 0.93–0.94), rectum (DSC 0.87–0.88), and sigmoid colon (approximately 0.80).

Other MRI‐only investigations demonstrated the progression from conventional machine learning approaches toward modern deep learning frameworks. Lu et al. [21] proposed a Bayesian framework integrating deformable registration, segmentation, and tumour detection, achieving tumour DSC values of approximately 0.78–0.82 across serial MRI examinations. Rouhi et al. compared multiple deep learning architectures and reported that an ensemble 2D SegResNet achieved the best performance with DSC of 0.72 ± 0.16 and incorporated a failure detection mechanism with 0.86 accuracy. Torheim et al. applied voxel‐based machine learning using multiparametric MRI and achieved an area under the receiver operating characteristic curve (AUC) up to 0.87, although DSC remained limited (up to 0.44), highlighting the difficulty of accurate tumour boundary delineation using conventional machine learning approaches.

MRI‐based studies also evaluated clinical workflow integration and generalizability. Simões et al. demonstrated that deep learning auto‐segmentation for MRI‐guided brachytherapy could reduce editing requirements, with patient‐specific models requiring less correction time compared with population‐based models. Transfer learning approaches were explored by Lin et al., who demonstrated improved generalizability from cervical cancer segmentation toward broader uterine malignancies, increasing DSC performance from 0.43 to 0.70 with limited additional data. Similarly, Ni et al. showed that transfer learning improved OAR segmentation across different scanners and imaging environments.

Additional MRI‐only studies further confirmed the potential of advanced architectures. Kano et al. combined 2D and 3D U‐Net models for diffusion‐weighted MRI segmentation, achieving a median DSC of 0.83 and HD of 4.7 mm. Leung et al. developed Masked‐Net and Dilated Masked‐Net models with transfer learning for 3D MRI segmentation, achieving an average DSC of 0.79 across tumours and OARs. Hodneland et al. evaluated a 3D residual U‐Net for whole‐volume tumour segmentation on multiparametric MRI, reporting median DSC values of approximately 0.60–0.81 depending on reference comparison. Shi et al. and Zhu et al. further demonstrated the potential of transformer‐based architectures, with TransUNet achieving DSC of 0.763 and CNN‐transformer hybrid models achieving DSC values of approximately 0.82–0.83. Overall, MRI‐only studies demonstrate that AI models can achieve clinically promising segmentation accuracy, particularly for larger tumours and well‐defined anatomical structures. However, performance remains variable for small, infiltrative, or treatment‐responsive tumours, and most studies remain limited by retrospective designs, single‐centre datasets, and insufficient external validation.

### 3.6 Multimodal Segmentation Studies

A smaller subset of studies investigated imaging strategies that incorporated MRI with additional modalities such as CT or PET, while one study evaluated CT‐only segmentation as complementary non‐MRI evidence. These studies represent emerging AI‐assisted approaches that aim to improve segmentation by combining complementary anatomical or functional information. However, because these approaches incorporate additional imaging information, their reported performance cannot be directly compared with MRI‐only segmentation models. Cao et al. developed a dual‐path asymmetric CNN using preimplant T2‐weighted MRI combined with postimplant CT for HR‐CTV segmentation in brachytherapy patients. The model achieved a DSC of 0.76 ± 0.06, sensitivity of 0.81, precision of 0.83, and HD95 of approximately 6 mm. Although multimodal information improved anatomical representation, the study was limited by small sample size and lack of external validation. Sun et al. investigated a CT‐to‐MR synthesis framework combined with multimodal segmentation using AGSC‐GAN and a ResNet‐based fully convolutional network. The approach achieved a DSC of 0.947, IOU of 0.904, and HD of 4.72 mm for clinical target volume segmentation. Despite strong reported performance, the clinical interpretation should consider that generated MRI information and multimodal fusion introduce additional computational steps and may limit direct comparison with conventional MRI‐only workflows. Liu et al. evaluated PET/MRI‐based cervical tumour segmentation using a parallel encoder U‐Net incorporating separate PET and MRI pathways. The multimodal model achieved DSC values of 0.73 for 3D segmentation and 0.87 for 2D segmentation, outperforming single‐modality approaches. However, the study remained limited by single‐center data and absence of external validation. Xue et al. applied a 3D Prompt‐ResUNet framework using CT and MRI data for HR‐CTV and OAR segmentation in cervical brachytherapy. The model achieved DSC values of approximately 0.92 for HR‐CTV and bladder, and 0.87 for rectum, demonstrating the potential benefit of combining anatomical information from multiple imaging modalities. Rigaud et al. investigated deep learning segmentation using planning CT for adaptive radiotherapy. The model achieved mean DSC values of 0.87–0.88 in internal validation and 0.78–0.79 in external evaluation, with clinically acceptable dose distributions. However, because this study relied on CT rather than MRI‐based segmentation, its findings should be interpreted as complementary evidence rather than direct evidence for MRI segmentation performance. Collectively, multimodal studies demonstrate promising results and highlight the potential value of integrating complementary imaging information. Nevertheless, these approaches require further validation to determine whether their additional complexity translates into meaningful clinical advantages over MRI‐only AI segmentation workflows.

### 3.7 Tumour Segmentation on MRI: Core Architectures and Performance Comparisons

A predominant focus across studies is the segmentation of gross tumour volume (GTV) or high‐risk clinical target volume (HR‐CTV) on T2‐weighted MRI, often enhanced by diffusion‐weighted imaging (DWI) and apparent diffusion coefficient (ADC) maps. Kalantar et al. introduced a multi‐head dilated DL framework to mitigate spatial misalignment in multiparametric MRI, achieving a median DSC of 0.823, which surpassed the conventional multi‐channel residual U‐Net's DSC of 0.788. This approach emphasized the dominant contributions of T2‐weighted MRI and ADC, while noting limited impact from b1000 DWI. Similarly, Lin et al. developed a triple‐channel U‐Net using b0, b1000, and ADC images, yielding a DSC of 0.82 and sensitivity of 0.89, with strong reproducibility in first‐order ADC radiomics features [15]. In contrast, Lu et al. targeted small cervical tumours with an augmented multiscale network (AugMS‐Net), incorporating a 3D Res2Net module and deep multiscale supervision, resulting in superior performance over baseline 3D networks and a 20% reduction in model parameters [21]. Hodneland et al. employed a 3D U‐Net for whole‐volume tumour segmentation on multiparametric MRI, attaining a DSC around 0.60 when compared to individual radiologists, though inter‐rater agreement among experts was higher [30]. Kano et al. combined 2D and 3D U‐Net frameworks on DWI, achieving a median DSC of 0.83 and HD of 4.7 mm, with better accuracy for larger, homogeneous tumours [31]. Rouhi et al. compared multiple 2D and 3D architectures, finding that an ensemble 2D SegResNet outperformed others with a DSC of 0.72 and surface DSC of 0.66, advocating for 2D models on 2D MRI sequences [22]. Torheim et al. utilized voxel‐wise classification with Fisher's Linear Discriminant Analysis on multiparametric MRI, reaching a DSC up to 0.44 and AUC up to 0.87. Shi et al. advanced this with a TransUnet model integrating CNNs and transformers, achieving a DSC of 0.7628 across multi‐directional T2‐weighted images [23]. Zhu et al. further refined transformer integration in a CNN‐Transformer (CoTr) model, yielding DSCs of 0.83 for contrast‐enhanced T1‐weighted and 0.82 for T2‐weighted images [43]. Xia et al. proposed an end‐to‐end Cer‐ConvN3Unet pipeline for multiparametric MRI, with DSCs of 0.83 for DWI, 0.79 for T2‐weighted, and 0.74 for contrast‐enhanced T1‐weighted, demonstrating high detection accuracy of 93% and reduced delineation time [40]. Leung et al. addressed data limitations with Masked‐Net and dilated Masked‐Net models using transfer learning and positional Dice loss, achieving an average DSC of 0.79 across structures, particularly for variable anatomies like the bladder and combined cervix‐GTV‐uterus [32].

Breto et al. evaluated a Mask R‐CNN for GTV and organs at risk (OARs) on planning and daily MRI, with DSCs above 0.8 for well‐defined organs like rectum and bladder, but around 0.6–0.7 for GTV and deformable structures [29]. Rodríguez Outeiral et al. applied nnU‐Net for GTV on brachytherapy MRI, attaining a median DSC of 0.73 with minimal dosimetric differences [38]. Wang et al. used a 3D‐CNN for multimodal MRI, achieving DSCs of 0.78 for whole tumour, 0.64 for core tumour, and 0.71 for enhanced tumour, with diagnostic accuracy of 93.11% [28]. Earlier work by Wu et al. introduced an iterative active deformational method, yielding a Jaccard similarity of 0.71 for preradiation tumours, though accuracy declined with tumour regression [27]. Lu et al. (from 2012) proposed a Bayesian‐based unified framework for serial MRI, maintaining DSCs around 0.78–0.82 despite deformation and regression.

### 3.8 Organs at Risk and Multimodal Extensions

Several studies extended segmentation to OARs alongside targets, enhancing brachytherapy and radiotherapy planning. Kim et al. developed a dual convolution‐transformer UNet (DCT‐UNet) with a coarse‐to‐fine strategy, achieving DSCs of 0.93 for bladder, 0.79 for rectum, 0.66 for sigmoid, and 0.74 for HR‐CTV, with computation times of 13 s per case. Zabihollahy et al. [18] presented an anatomy‐guided cascaded approach for clinical target volume (CTV), with a mean DSC of 0.85 and 95th percentile HD of 3.70 mm, including uncertainty maps. Zabihollahy et al. [20] focused on multi‐organ segmentation with a coarse‐to‐fine 3D U‐Net and Dense U‐Net, yielding DSCs of 0.93 for bladder, 0.87–0.88 for rectum, and 0.80 for sigmoid. Yoganathan et al. compared 2D and 2.5D models, favouring 2.5D InceptionResNetv2 for better dosimetric agreement in GTV and HR‐CTV [18, 20]. Multimodal integrations incorporated CT or PET. Cao et al. used a dual‐path asymmetric CNN for HR‐CTV on postimplant CT with preimplant MRI, achieving a DSC of 0.76 and HD around 6 mm [19]. Sun et al. employed an attention‐guided GAN for CT‐to‐MR generation and multimodal fusion, yielding a DSC of 0.947 [26]. Liu et al. proposed a parallel encoder U‐Net (PEU‐Net) for PET/MRI, outperforming baselines in DSC and HD [34]. Xue et al. introduced 3D Prompt‐ResUNet for HRCTV and OARs on CT/MRI, with DSCs around 0.92 for HRCTV and bladder [41]. Rigaud et al. validated 2D DeepLabV3+ and 3D U‐Net on CT for adaptive radiotherapy, meeting clinical dose criteria [37]. Liang et al. integrated DL with wireless sensor networks for MRI, achieving accuracies of 98%–99% [33].

### 3.9 Generalizability, Transfer Learning, and Clinical Integration

Transfer learning emerged as a strategy for generalizability. Lin et al. applied it to extend cervical models to uterine malignancies, boosting DSCs from 0.43 to 0.70 with limited data [25]. Ni et al. fine‐tuned nnU‐Net for OARs across scanners, restoring performance with minimal scans [35]. Ni et al. introduced distance‐penalized training and weighted DSC, improving near‐target accuracy and dosimetric correlation [36]. Clinical impact was assessed in adaptive settings. Simões et al. found patient‐specific auto‐segmentations reduced editing time in brachytherapy [24]. Rouhi et al. added radiomics‐based failure detection, achieving 0.86 accuracy for identifying poor segmentations [22].

### 3.10 Strengths, Limitations, and Innovations

Across studies, DL models generally demonstrated promising segmentation performance and potential workflow‐efficiency benefits, with DSCs typically ranging from 0.60 to 0.93 depending on structure complexity and tumour size. Innovations like dilated convolutions [14], transformers [17, 39, 43], and multimodal fusion [26, 34] enhanced boundary awareness and robustness. However, challenges persist in small or regressing tumours [27, 31], deformable OARs [29], and multicenter generalizability, often requiring transfer learning [15, 25, 35, 36]. Limitations include dataset sizes and reliance on single‐institution data, potentially limiting external validity. Few studies provided uncertainty quantification [18] or failure detection [22], which are crucial for clinical safety.

## 4 Discussion

### 4.1 Clinical Implications of AI‐Assisted Segmentation

The findings of this systematic review demonstrate that AI‐based segmentation approaches have the potential to improve efficiency, reproducibility, and standardization in cervical cancer imaging workflows. Automated segmentation may reduce the considerable time required for manual contour delineation and minimize inter‐observer variability, which remains a major challenge in radiotherapy planning and brachytherapy [14, 17, 18, 20, 25, 31, 39, 40, 43]. Accurate delineation of gross tumour volume (GTV), clinical target volume (CTV), and organs at risk (OARs) is essential for precise radiation delivery. AI‐assisted segmentation could facilitate more consistent target definition, improve treatment planning efficiency, and support adaptive radiotherapy workflows, particularly in settings where experienced contouring specialists are limited. In MRI‐guided brachytherapy, automated segmentation may assist clinicians by providing initial contours that can be reviewed and edited rather than generated manually from the beginning. This approach may reduce contouring workload while maintaining clinician oversight. Beyond accuracy metrics, the clinical value of AI should be evaluated through reductions in segmentation time, improved workflow consistency, and integration into existing radiotherapy planning systems. Several studies also suggest potential reductions in contour editing time and improved workflow efficiency; however, few evaluated these benefits prospectively in routine clinical environments. Similarly, although some studies reported limited dosimetric differences after AI‐assisted contouring, the effect of segmentation errors on treatment planning decisions remains insufficiently investigated. By providing standardized initial segmentations, AI systems may reduce variability between observers and institutions; however, expert review remains necessary because anatomical complexity and tumour heterogeneity can affect model performance. These findings suggest that AI‐assisted segmentation may reduce contouring time and improve consistency compared with manual workflows in selected settings, particularly when leveraging multiparametric data; however, the available evidence remains limited and heterogeneous, and expert review remains necessary. The QUADAS‐2 assessment highlights important methodological limitations that should be considered when interpreting reported segmentation performance. Although many models achieved high DSC values, frequent concerns regarding single‐center datasets, lack of independent external validation, and variability in reference annotations may lead to optimistic performance estimates and limit clinical generalization.

### 4.2 Challenges for Clinical Implementation

Despite the promising segmentation performance demonstrated by AI‐based approaches, several important barriers must be addressed before these systems can be translated into routine clinical practice. A major limitation of the current evidence base is the lack of robust external validation. Most reported models were developed and evaluated using single‐centre datasets, which limits confidence regarding their performance across different institutions, imaging scanners, acquisition protocols, and patient populations. Models trained on highly specific institutional datasets may perform well under controlled conditions but may demonstrate reduced accuracy when applied to external clinical environments with different imaging characteristics or patient demographics. Therefore, independent multicenter validation studies are essential to determine the true generalizability and reliability of AI‐assisted segmentation systems. In addition, regulatory and quality assurance considerations represent critical steps toward clinical implementation. Translation of AI segmentation tools into healthcare workflows requires appropriate regulatory evaluation, standardized validation procedures, transparent reporting of model development and performance, and continuous monitoring after deployment. Unlike conventional imaging software, AI systems may evolve or demonstrate variable behaviour when exposed to new clinical data, making post‐deployment surveillance and quality control essential to ensure patient safety. Another important challenge is model drift, which may occur when changes in MRI acquisition protocols, scanner hardware, reconstruction techniques, or patient characteristics alter the distribution of input data compared with the original training dataset. Such changes can gradually reduce model performance over time and highlight the need for periodic recalibration, performance assessment, and potential model updating before widespread clinical adoption. Furthermore, dataset bias remains a significant concern, as many existing AI systems are trained using limited demographic and institutional datasets that may not adequately represent the diversity of real‐world cervical cancer populations (Table S7). Consequently, models may exhibit reduced performance in underrepresented groups, emphasizing the importance of developing diverse, inclusive datasets and evaluating algorithm performance across different patient populations. Addressing these challenges will be essential to ensure that AI‐assisted segmentation tools achieve safe, equitable, and clinically meaningful integration into cervical cancer management.

### 4.3 Innovations in Model Architectures and Techniques

Recent advances in AI architectures have improved automated segmentation performance through enhanced feature extraction, multimodal integration, and improved representation of complex anatomical structures. Across the included studies, AI models demonstrated promising segmentation performance, with DSC values varying according to anatomical target, imaging protocol, and model architecture. Higher performance was generally observed for well‐defined structures and larger tumour regions, whereas more challenging targets showed reduced accuracy [14, 19, 21, 22, 23, 27, 28, 29, 30, 38, 39, 40, 43]. The evolution from conventional convolutional neural network (CNN)‐based approaches toward advanced U‐Net variants, transformer‐based models, and hybrid architectures has improved the ability of AI systems to capture both local image features and broader contextual information. Transfer learning and multimodal fusion strategies have also emerged as important approaches to improve model robustness, particularly when available training datasets are limited. However, the increasing complexity of these models introduces additional challenges, including computational requirements, interpretability limitations, and the need for more comprehensive validation before clinical deployment.

### 4.4 Methodological Challenges and Current Limitations of AI Segmentation Research

Although AI approaches have demonstrated encouraging capabilities, several methodological challenges limit the interpretation and clinical translation of current evidence. A major issue is the frequent use of relatively small datasets and retrospective single‐institution cohorts. Limited sample sizes increase the risk of overfitting and may result in overly optimistic estimates of model performance. Furthermore, differences in MRI acquisition protocols, imaging sequences, preprocessing strategies, and annotation procedures introduce substantial heterogeneity between studies, making direct comparison of reported outcomes difficult. The complexity of cervical cancer segmentation itself also influences AI performance. Models generally demonstrate stronger performance for larger and clearly defined anatomical structures, whereas segmentation of small tumours, infiltrative lesions, regressing tumours, or deformable anatomical structures remains challenging. These limitations emphasize that AI performance depends not only on algorithm architecture but also on image quality, tumour characteristics, annotation quality, and the specific clinical task being addressed.

A major challenge identified in this review was substantial heterogeneity among included studies. Differences in patient populations, tumour stage, MRI acquisition protocols, segmentation targets, annotation strategies, validation designs, and AI architectures limit direct comparison of reported performance metrics. For example, DSC values obtained for large, well‐defined organs at risk cannot be directly compared with those achieved for small, infiltrative, or treatment‐regressing tumours. Furthermore, variation in reference standards and the absence of standardized evaluation frameworks may contribute to uncertainty regarding the true clinical performance of reported models. Therefore, although individual studies demonstrate promising accuracy, the overall reliability and generalizability of the evidence remain limited.

The QUADAS‐2 assessment further highlighted methodological concerns related to patient selection, reference standards, and lack of independent validation. Although expert manual contours remain the accepted reference standard, variability between annotators and the absence of standardized consensus annotations in many studies may introduce uncertainty into model evaluation. Additionally, the limited reporting of uncertainty estimation and failure detection mechanisms remains an important barrier to safe clinical implementation.

### 4.5 Explainability and Uncertainty‐Aware AI

Explainability and uncertainty estimation represent essential components for safe clinical deployment of AI segmentation systems. Although some studies incorporated uncertainty maps or failure detection strategies, most investigated models provide deterministic predictions without quantifying confidence. Future systems should integrate uncertainty estimation, interpretable attention mechanisms, and automated quality‐control procedures to allow clinicians to identify unreliable predictions before clinical use.

### 4.6 Future Research Priorities

Future research should focus on addressing the current limitations of AI‐assisted cervical cancer segmentation through large‐scale, multicentre, and prospectively collected datasets. Such datasets should include diverse patient populations, imaging systems, and clinical environments to improve model robustness and external validity. Standardization of reporting practices and evaluation metrics is also essential to facilitate meaningful comparison between AI systems. Future studies should move beyond reporting segmentation accuracy alone and incorporate clinically relevant outcomes, including contouring time reduction, treatment planning efficiency, dosimetric impact, and patient‐centered outcomes [42]. Explainable AI (XAI) and uncertainty estimation should become integral components of future AI development. Providing clinicians with information regarding model confidence, segmentation reliability, and potential failure cases may improve trust and allow safer clinical decision‐making. Uncertainty‐aware systems and automated quality‐control mechanisms could help identify cases requiring additional expert review.

Finally, prospective clinical trials are needed to determine whether AI‐assisted segmentation provides measurable benefits compared with conventional workflows. Evaluation of real‐world implementation, cost‐effectiveness, workflow integration, and long‐term performance monitoring will be necessary before AI segmentation can become an established component of cervical cancer management.

## 5 Conclusion

This systematic review demonstrates that ML and DL methods—particularly CNN‐ and U‐Net–based architectures and emerging transformer‐enhanced models—have substantially advanced automated cervical cancer segmentation on MRI, with promising segmentation performance and potential workflow‐efficiency benefits compared with manual contouring. However, translation into clinical practice requires stronger external validation, standardized evaluation frameworks, and safety‐oriented AI strategies including uncertainty estimation and failure detection. Future research should prioritize large, multi‐institutional validation cohorts, standardized reporting of segmentation and dosimetric outcomes, and clinically oriented safeguards such as uncertainty estimation and failure detection. With these advances, AI‐driven segmentation may enhance radiotherapy planning, brachytherapy workflows, and longitudinal tumour monitoring, ultimately supporting more consistent and scalable cervical cancer care.

## Funding

The authors have nothing to report.

## Ethics Statement

This study is a systematic review and meta‐analysis of previously published studies and did not involve the recruitment of new participants or collection of identifiable individual‐level data.

## Consent

The authors have nothing to report.

## Conflicts of Interest

The authors declare no conflicts of interest.

## Supporting information


**Table S1:** Study‐level risk‐of‐bias assessment using the QUADAS‐2 tool.
**Table S2:** Applicability assessment of the included studies using the QUADAS‐2 tool.
**Table S3:** Detailed domain‐specific justifications for the QUADAS‐2 risk‐of‐bias assessment.
**Table S4:** PICO framework and database‐specific literature search strategy.
**Table S5:** Reasons for exclusion of full‐text articles during eligibility assessment.
**Table S6:** Comparative summary of AI architectures used for cervical cancer segmentation.
**Table S7:** Clinical readiness assessment of AI segmentation studies.

## Data Availability

All data analysed in this study were derived from included published articles and their Supporting Information. The extracted dataset and analytic code are available from the corresponding author upon reasonable request.
