# Supplementary material for: Deep Learning and Machine Learning Algorithms for Cervical Cancer Segmentation on MRI: A Systematic Review
Source: J Med Radiat Sci. 2026 Sep 27:10.1002/jmrs.70127. Online ahead of print. doi: 10.1002/jmrs.70127 (PMC13617261; doi:10.1002/jmrs.70127)
Supplement: Supplementary file 1 [file JMRS-9999-0-s001.docx]

**Supporting Information**

**Supplementary Table S1. Study-level risk-of-bias assessment using the QUADAS-2 tool.**

| **Study** | **Patient Selection** | **Index Test** | **Reference Standard** | **Flow and Timing** | **Overall Risk of Bias** |
| --- | --- | --- | --- | --- | --- |
| Kalantar et al., 2023 [14] | Low | Low | High | Low | High |
| Lin et al., 2019 [15] | Low | Some concerns | Low | Low | Low |
| Lu et al., 2021 [16] | Some concerns | Some concerns | Some concerns | Low | Some concerns |
| Sun et al., 2024 [26] | Some concerns | Some concerns | Low | Low | Low |
| Kim et al., 2024 [17] | Low | Low | Low | Low | Some concerns |
| Zabihollahy et al., 2022 [18] | Some concerns | Some concerns | Some concerns | Low | Some concerns |
| Cao et al., 2022 [19] | Some concerns | Some concerns | Some concerns | Low | Some concerns |
| Zabihollahy et al., 2021 [20] | Some concerns | Some concerns | Some concerns | Low | Some concerns |
| Lu et al., 2012 [21] | High | Some concerns | Some concerns | Low | High |
| Rouhi et al., 2024 [22] | Low | Low | Some concerns | Low | Low |
| Torheim et al., 2017 [23] | Some concerns | Some concerns | Low | Low | Some concerns |
| Simões et al., 2024 [24] | Some concerns | Some concerns | Some concerns | Low | Some concerns |
| Lin et al., 2023 [25] | Low | Low | Low | Low | Low |
| Wu et al., 2008 [27] | Some concerns | Some concerns | Low | Low | Some concerns |
| Wang et al., 2021 [28] | High | High | Some concerns | High | High |
| Breto et al. (2022) [29] | High | Low | High | Low | High |
| Hodneland et al. (2022) [30] | High | Low | Low | Low | High |
| Kano et al. (2021) [31] | High | Low | High | Low | High |
| Leung et al. (2024) [32] | High | Low | High | Low | High |
| Liang et al. (2019) [33] | High | High | High | High | High |
| Liu et al. (2025) [34] | High | Low | High | Low | High |
| Ni et al. (2024) [35] | Some concerns | Low | Some concerns | Low | Some concerns |
| Ni et al. (2025) [36] | Some concerns | Low | Some concerns | Low | Some concerns |
| Rigaud et al., 2021 [37] | Low | Some concerns | Some concerns | Low | Some concerns |
| Rodríguez Outeiral et al., 2023 [43] | Some concerns | Some concerns | Some concerns | Low | Some concerns |
| Shi et al., 2025 [38] | Some concerns | Some concerns | Some concerns | Low | Some concerns |
| Xia et al., 2025 [39] | Low | Low | Low | Low | Low |
| Xue et al., 2024 [40] | Some concerns | Some concerns | Low | Low | Some concerns |
| Yoganathan et al., 2022 [41] | Some concerns | Some concerns | Some concerns | Low | Some concerns |
| Zhu et al., 2024 [42] | Some concerns | Some concerns | Low | Low | Some concerns |

**Supplementary Table S2. Applicability assessment of the included studies using the QUADAS-2 tool.**

| **Study** | **Patient Selection** | **Index Test** | **Reference Standard** | **Flow and Timing** | **Overall Applicability Concern** |
| --- | --- | --- | --- | --- | --- |
| Kalantar et al., 2023 [14] | Low | Low | Low | Low | Low |
| Lin et al., 2019 [15] | Low | Low | Low | Low | Low |
| Lu et al., 2021 [16] | Some concerns | Low | Low | Low | Some concerns |
| Kim et al., 2024 [17] | Low | Low | Low | Low | Low |
| Zabihollahy et al., 2022 [18] | Low | Low | Low | Low | Low |
| Cao et al., 2022 [19] | Low | Low | Low | Low | Low |
| Zabihollahy et al., 2021 [20] | Low | Low | Low | Low | Low |
| Lu et al., 2012 [21] | Some concerns | Low | Low | Low | Some concerns |
| Rouhi et al., 2024 [22] | Low | Low | Low | Low | Low |
| Torheim et al., 2017 [23] | Low | Low | Low | Low | Low |
| Simões et al., 2024 [24] | Low | Low | Low | Low | Low |
| Lin et al., 2023 [25] | Low | Low | Low | Low | Low |
| Wu et al., 2008 [27] | Low | Some concerns | Low | Low | Some concerns |
| Wang et al., 2021 [28] | High | Low | Low | Low | High |
| Breto et al. (2022) [29] | High | Low | Low | Low | High |
| Hodneland et al. (2022) [30] | Some concerns | Low | Low | Low | Some concerns |
| Kano et al. (2021) [31] | Some concerns | Low | Low | Low | Some concerns |
| Leung et al. (2024) [32] | Some concerns | Low | Low | Low | Some concerns |
| Liang et al. (2019) [33] | High | High | High | High | High |
| Liu et al. (2025) [34] | Some concerns | Low | Low | No information | Some concerns |
| Ni et al. (2024) [35] | Low | Low | Low | Low | Low |
| Ni et al. (2025) [36] | Low | Low | Low | Low | Low |
| Rigaud et al., 2021 [37] | Low | Low | Low | Low | Low |
| Rodríguez Outeiral et al., 2023 [43] | Low | Low | Low | Low | Low |
| Shi et al., 2025 [38] | Low | Some concerns | Low | Low | Some concerns |
| Xia et al., 2025 [39] | Low | Low | Low | Low | Low |
| Xue et al., 2024 [40] | Low | Low | Low | Low | Low |
| Yoganathan et al., 2022 [41] | Low | Low | Low | Low | Low |
| Zhu et al., 2024 [42] | Low | Low | Low | Low | Low |

**Supplementary Table S3. Detailed domain-specific justifications for the QUADAS-2 risk-of-bias assessment.**

| **Study** | **Domain** | **Risk of Bias** | **Justification (RoB)** | **Applicability Concern** |
| --- | --- | --- | --- | --- |
| Kalantar et al., 2023 [14] | 1. Patient Selection | Low | Consecutive retrospective cohort of 207 patients with locally advanced cervical cancer. Inclusion criteria clearly defined; no inappropriate exclusions reported. | Low |
| Kalantar et al., 2023 [14] | 2. Index Test (Deep Learning Model) | Low | Index test was developed and evaluated using supervised learning with predefined architecture; however, model thresholds and training decisions were optimized on the same dataset (risk of overfitting). No fully external validation cohort. | Low |
| Kalantar et al., 2023 [14] | 3. Reference Standard (Ground Truth Segmentation) | High | Tumor contours were delineated by a single clinician with 3 years’ experience; no consensus contours or inter-reader variability assessment, introducing potential subjective bias. | Low |
| Kalantar et al., 2023 [14] | 4. Flow and Timing | Low | All patients underwent the same imaging protocol; all cases were included in training, validation, and testing without differential verification. Appropriate data splitting reported. | Low |
| Lin et al., 2019 [15] | 1. Patient Selection | Low | Consecutive retrospective cohort with clearly defined inclusion and exclusion criteria. Independent training (n=144) and testing (n=25) datasets used, reducing selection bias. | Low |
| Lin et al., 2019 [15] | 2. Index Test (Deep Learning Model) | Moderate | Model development involved optimization and selection of input channels on the training dataset; probability threshold fixed at 0.5 but no external (multi-center) validation performed, raising risk of overfitting. | Low |
| Lin et al., 2019 [15] | 3. Reference Standard (Ground Truth Segmentation) | Low | Manual tumor contours were delineated by an experienced radiologist and independently verified by a second expert, both blinded to outcomes, reducing subjectivity bias. | Low |
| Lin et al., 2019 [15] | 4. Flow and Timing | Low | All patients received the same reference standard and index test; no partial verification or unexplained exclusions. Imaging-to-reference interval appropriate (median 13 days). | Low |
| Lu et al., 2021 [16] | 1. Patient Selection | Moderate | The study uses retrospective datasets (CeTS cervical MRI dataset and LiTS CT dataset). Details on consecutive enrollment and exclusion criteria for the cervical MRI cohort are limited, raising potential selection bias. | Moderate |
| Lu et al., 2021 [16] | 2. Index Test (AugMS-Net segmentation model) | Moderate | Model architecture, training, and evaluation are clearly described; however, hyperparameters and architectural choices were optimized on the available datasets, with no external multi-center clinical validation, increasing overfitting risk. | Low |
| Lu et al., 2021 [16] | 3. Reference Standard (Ground-Truth Segmentation) | Moderate | Ground-truth tumor annotations are derived from expert manual segmentations, but the number of annotators, consensus strategy, and inter-observer variability are not clearly reported. | Low |
| Lu et al., 2021 [16] | 4. Flow and Timing | Low | All cases appear to receive the same reference standard and index test, with no partial verification or differential flow reported. | Low |
| Kim et al., 2024 [17] | 1. Patient Selection | Low | Patients undergoing cervical cancer brachytherapy MRI were used; inclusion aligns with routine clinical workflow. No inappropriate exclusions reported, and study objective matches dataset. | Low |
| Kim et al., 2024 [17] | 2. Index Test (DCT-UNet model) | Moderate | Model architecture, training strategy, and size-adaptive approach are clearly defined; however, model development and evaluation were performed within the same institutional dataset, with no external multicenter validation. | Low |
| Kim et al., 2024 [17] | 3. Reference Standard (Ground-Truth Contours) | Low | OAR contours were drawn by the attending radiation oncologist. HR-CTV reference standard was based on consensus contours derived from four contour sets, with explicit inter- and intra-observer variability analysis, minimizing subjectivity bias. | Low |
| Kim et al., 2024 [17] | 4. Flow and Timing | Low | All cases received the same index test and reference standard; no partial verification or differential flow. Imaging and contouring reflect standard clinical timing. | Low |
| Zabihollahy et al., 2022 [18] | 1. Patient Selection | Moderate | Retrospective, single-institution dataset (213 MRI scans from 125 patients). Consecutive inclusion is not explicitly stated and multiple scans per patient were included, which may introduce selection bias. | Low |
| Zabihollahy et al., 2022 [18] | 2. Index Test (CNN-based CTV segmentation) | Moderate | Model architecture and training are clearly described; however, model development and evaluation were performed on data from the same institution, with no external or multicenter validation, raising overfitting and optimism bias concerns. | Low |
| Zabihollahy et al., 2022 [18] | 3. Reference Standard (Manual CTV contours) | Moderate | Ground truth contours were generated by residents and reviewed/corrected by a single attending radiation oncologist. No multi-observer consensus or inter-observer variability analysis was performed, and CTV lacks a true gold standard. | Low |
| Zabihollahy et al., 2022 [18] | 4. Flow and Timing | Low | All cases received the same index test and reference standard; clear separation of training, validation, and independent test sets with no partial verification or unexplained exclusions. | Low |
| Cao et al., 2022 [19] | 1. Patient Selection | Moderate | Retrospective, single-institution cohort (65 patients). Consecutive inclusion is not explicitly stated and sample size is relatively small, which may introduce selection bias. | Low |
| Cao et al., 2022 [19] | 2. Index Test (Dual-path CNN using MR + CT) | Moderate | Model architecture and training are clearly described, but development and testing were performed within the same institutional dataset using cross-validation, with no external or multicenter validation, increasing risk of overfitting. | Low |
| Cao et al., 2022 [19] | 3. Reference Standard (Manual HR-CTV contours) | Moderate | HR-CTV contours were primarily verified by a single experienced radiation oncologist; although inter- and intra-observer variability analyses were performed, the reference standard is not a full multi-expert consensus and HR-CTV lacks a true gold standard. | Low |
| Cao et al., 2022 [19] | 4. Flow and Timing | Low | All patients received the same index test and reference standard. Clear data flow with defined training, validation, and testing splits; no partial verification or unexplained exclusions. | Low |
| Zabihollahy et al., 2021 [20] | 1. Patient Selection | Moderate | Retrospective, single-institution dataset (181 patients; 283 MR scans). Consecutive inclusion was not explicitly stated, and multiple scans per patient were included, which may introduce selection bias. | Low |
| Zabihollahy et al., 2021 [20] | 2. Index Test (Coarse-to-fine CNN model) | Moderate | Model architecture and training are clearly described, but model development, tuning, and testing were performed on data from the same institution. No external or multicenter validation was conducted, increasing risk of overfitting. | Low |
| Zabihollahy et al., 2021 [20] | 3. Reference Standard (Manual OAR contours) | Moderate | Ground truth contours were created by radiation oncology residents and reviewed by an expert radiation oncologist. No multi-observer consensus or formal inter-observer variability analysis was performed. | Low |
| Zabihollahy et al., 2021 [20] | 4. Flow and Timing | Low | All cases underwent the same index test and reference standard, with clear training, fine-tuning, and independent testing splits. No partial verification or unexplained exclusions were reported. | Low |
| Lu et al., 2012 [21] | 1. Patient Selection | High | Very small cohort (6 patients, 36 MR scans). Highly selective sample from a single institution; no consecutive or representative sampling. Repeated scans per patient increase dependency and risk of selection bias. | Moderate |
| Lu et al., 2012 [21] | 2. Index Test (Segmentation / Registration / Tumor Detection Framework) | Moderate | Index test was developed and evaluated on the same dataset using leave-one-patient-out validation. Algorithm thresholds (e.g., tumor probability cutoff) were chosen empirically, introducing optimization bias. | Low |
| Lu et al., 2012 [21] | 3. Reference Standard (Manual Delineation) | Moderate | Manual contours were created by a qualified clinician and approved by a radiologist, but no multi-observer consensus or inter-observer variability analysis was performed. Tumor detection lacks a true gold standard. | Low |
| Lu et al., 2012 [21] | 4. Flow and Timing | Low | All patients received the same index test and reference standard at each time point. No partial verification or unexplained exclusions. Imaging intervals reflect routine weekly treatment workflow. | Low |
| Rouhi et al., 2024 [22] | 1. Patient Selection | Low | Large, well-described cohorts: retrospective multicenter training cohort (115 patients, 30 scanners) and an independent prospective multicenter test cohort (51 patients, 7 scanners). Clear separation of training and testing reduces selection and spectrum bias. | Low |
| Rouhi et al., 2024 [22] | 2. Index Test (DL-based GTV segmentation + failure detection) | Low | Multiple predefined DL architectures evaluated using cross-validation; final model tested on an external prospective cohort. Failure-detection thresholds defined using training data only, limiting optimism bias. | Low |
| Rouhi et al., 2024 [22] | 3. Reference Standard (Manual GTV contours) | Moderate | GTV contours were produced by expert radiation oncologists, but no formal multi-observer consensus contour set was used as ground truth; GTV inherently lacks a true gold standard. | Low |
| Rouhi et al., 2024 [22] | 4. Flow and Timing | Low | All patients received the same reference standard and index test. Prospective test cohort reflects real-world timing and imaging variability; no partial verification or unexplained exclusions. | Low |
| Torheim et al., 2017 [23] | 1. Patient Selection | Moderate | Retrospective cohort of 78 patients from a single institution (MRI acquired 2001–2004). Consecutive enrollment is not explicitly stated, and only slices containing tumor were included, which may inflate performance and introduce selection bias. | Low |
| Torheim et al., 2017 [23] | 2. Index Test (Machine-learning voxel classification: LDA/QDA) | Moderate | Index test thresholds (50% probability cutoff) and feature combinations were selected during model development. Although leave-one-patient-out cross-validation was used, there was no independent external validation cohort. | Low |
| Torheim et al., 2017 [23] | 3. Reference Standard (Radiologist tumor contours) | Low | Tumor delineations were created by two experienced radiologists with consensus agreement, reducing subjectivity and reference standard bias. | Low |
| Torheim et al., 2017 [23] | 4. Flow and Timing | Low | All patients received the same index test and reference standard. Leave-one-out cross-validation ensured each patient was tested independently; no partial verification or unexplained exclusions were reported. | Low |
| Simões et al., 2024 [24] | 1. Patient Selection | Moderate | Retrospective single-center cohort of 28 patients. Consecutive inclusion is not explicitly stated and sample size is relatively small, which may limit representativeness. | Low |
| Simões et al., 2024 [24] | 2. Index Test (DL-based target auto-segmentation) | Moderate | Population-based and patient-specific models were evaluated retrospectively on the same institutional cohort. No independent external validation cohort was used, and manual editing was performed with awareness of the auto-segmentation, introducing potential automation bias. | Low |
| Simões et al., 2024 [24] | 3. Reference Standard (Clinical target delineations) | Moderate | Clinical contours were created and reviewed by radiation oncologists following GEC-ESTRO/ICRU guidelines; however, no multi-observer consensus reference standard was generated specifically for this study, and target volumes inherently lack a true gold standard. | Low |
| Simões et al., 2024 [24] | 4. Flow and Timing | Low | All patients received the same index tests and reference standard for the second fraction. Clear flow without partial verification or unexplained exclusions; timing reflects real clinical practice. | Low |
| Lin et al., 2023 [25] | 1. Patient Selection | Low | Large retrospective multicenter cohort with clearly defined inclusion and exclusion criteria. Consecutive uterine cases were screened (345 patients), and reasons for exclusion (no visible tumor, DW artifacts) were explicitly reported (flowchart, Fig. 2). Separate training and independent testing sets were used. | Low |
| Lin et al., 2023 [25] | 2. Index Test (DeepLab V3+ with transfer learning) | Moderate | Model architecture and training strategy were prespecified; however, multiple TL configurations and training sample sizes were explored within the same dataset, which may introduce model-selection and optimization bias. No prospective external validation cohort was used. | Low |
| Lin et al., 2023 [25] | 3. Reference Standard (Manual tumor segmentation) | Low | Ground-truth ROIs were delineated by two experienced gynecologic radiologists in consensus, blinded to clinical outcomes, minimizing subjectivity and reference standard bias. | Low |
| Lin et al., 2023 [25] | 4. Flow and Timing | Low | All patients underwent the same index test and reference standard. Clear separation of training, validation, and testing datasets with no partial verification or unexplained exclusions. | Low |
| Wu et al., 2008 [27] | 1. Patient Selection | Moderate | Retrospective cohort of 52 patients (121 MRI studies) with stage IIB–IVB disease. Consecutive inclusion is not explicitly stated, and patient attrition occurred across radiation stages, which may introduce selection bias. | Low |
| Wu et al., 2008 [27] | 2. Index Test (Iterative active deformational segmentation) | Moderate | Semi-automated method requires user initialization and interaction; parameters and stopping criteria are user-dependent and not blinded to reference contours, introducing potential performance and observer-related bias. No external validation cohort. | Moderate |
| Wu et al., 2008 [27] | 3. Reference Standard (Manual tumor delineation) | Low | Manual segmentations were performed by an experienced radiation oncologist (>10 years’ experience). This represents a credible clinical reference, although no multi-observer consensus or variability analysis was performed. | Low |
| Wu et al., 2008 [27] | 4. Flow and Timing | Low | All patients received the same index test and reference standard at each imaging stage. Timing of MRI relative to treatment stages was clearly described; no partial verification or unexplained exclusions. | Low |
| Wang et al., 2021 [28] | 1. Patient Selection | High | Small, single-center retrospective cohort (70 cervical cancer patients + 10 healthy controls). Case–control–like design with healthy controls is inappropriate for diagnostic accuracy studies and may inflate performance estimates. Consecutive inclusion not stated. | High |
| Wang et al., 2021 [28] | 2. Index Test (3D-CNN multimodal MRI algorithm) | High | Index test was developed and evaluated on the same dataset with no clear separation of training/validation/testing sets. Thresholds and model optimization were data-driven, and blinding to reference standard was not reported, indicating high risk of overfitting and optimism bias. | Low |
| Wang et al., 2021 [28] | 3. Reference Standard (Radiologist lesion delineation / pathology) | Moderate | Lesion regions were delineated by three radiologists, but consensus methodology and inter-observer variability analysis were not clearly reported. Pathology was used for diagnosis but not explicitly for pixel-level ground truth. | Low |
| Wang et al., 2021 [28] | 4. Flow and Timing | High | Unclear data flow: no explicit reporting of independent test set, repeated use of training data for performance evaluation, and no transparency on how radiologists’ annotations were split across development and testing phases. | Low |
| Breto et al. (2022) [29] | Patient Selection | High | Small, single-center cohort (n = 15) selected retrospectively from an institutional registry; no random or consecutive sampling and no clear inclusion/exclusion flow reported, increasing risk of selection bias. | High |
| Breto et al. (2022) [29] | Index Test (Deep Learning Segmentation Model) | Low | The Mask R-CNN model was clearly described, predefined, and evaluated using objective, standard metrics (DSC, Hausdorff distance) without post-hoc threshold optimization. | Low |
| Breto et al. (2022) [29] | Reference Standard (Manual Contouring) | Unclear | Manual contours were produced and reviewed by expert radiation oncologists, but inter-observer variability and blinding between contouring and model output were not formally quantified or reported. | Low |
| Breto et al. (2022) [29] | Flow and Timing | Low | All patients underwent the same imaging protocol, and both planning and per-fraction MRIs were analyzed consistently; no exclusions after enrollment were reported. | Low |
| Hodneland et al. (2022) [30] | Patient Selection | High | Retrospective design with selective inclusion (only patients with visible tumors on MRI confirmed by both radiologists); exclusion of very large tumors (>1000 mL) and poor-quality scans may introduce selection bias and spectrum bias. | Moderate |
| Hodneland et al. (2022) [30] | Index Test (DL Segmentation) | Low | Deep learning model and thresholding strategy were predefined and consistently applied; evaluation used objective metrics (DSC, HD) without data-driven threshold optimization on the test set. | Low |
| Hodneland et al. (2022) [30] | Reference Standard (Manual Segmentation) | Low | Manual segmentations were performed by experienced radiologists using standardized software, with blinding to clinical data; inter-rater agreement was explicitly quantified in the test cohort. | Low |
| Hodneland et al. (2022) [30] | Flow and Timing | Low | All included patients received the same reference standard and index test; clear separation of training/validation and independent test cohort; no differential verification or inappropriate exclusions after inclusion. | Low |
| Kano et al. (2021) [31] | Patient Selection | High | Retrospective, single-center study including 98 patients treated between 2005–2013; no consecutive or random sampling strategy reported, and patients were included based on availability of DWI, introducing potential selection and spectrum bias. | Moderate |
| Kano et al. (2021) [31] | Index Test (AI-based GTV Segmentation) | Low | The AI model (combined 2D/3D U-Net with predefined k-fold cross-validation and model averaging) was clearly specified before evaluation; objective performance metrics (DSC, HD) were used without post-hoc threshold tuning on the test data. | Low |
| Kano et al. (2021) [31] | Reference Standard (Manual GTV Contouring) | High | Reference contours were created by a single highly experienced radiation oncologist; absence of multiple annotators, inter-observer variability assessment, or blinding increases risk of reference standard bias. | Low |
| Kano et al. (2021) [31] | Flow and Timing | Low | All included patients underwent the same imaging protocol and reference standard; no partial verification or differential verification reported, and cross-validation ensured all cases were evaluated. | Low |
| Leung et al. (2024) [32] | Patient Selection | High | Retrospective study with a small cohort (23 patients, 52 MRI volumes) and repeated scans per patient; exclusion of four “outlier” cases from testing introduces selection bias and may inflate performance estimates. | Moderate |
| Leung et al. (2024) [32] | Index Test (Deep Learning Segmentation Model) | Low | The Masked-Net/DM-Net architectures, transfer learning strategy, and loss functions were pre-specified and evaluated using standard metrics (DSC, HD, MSD) without outcome-driven threshold adjustment. | Low |
| Leung et al. (2024) [32] | Reference Standard (Manual Contouring) | High | Manual contours were provided by a single experienced radiation oncologist; absence of multi-observer annotation, consensus contours, or blinding increases risk of reference standard bias. | Low |
| Leung et al. (2024) [32] | Flow and Timing | Low | All included MRI volumes underwent the same reference standard and index test, with consistent 5-fold cross-validation; timing between imaging and contouring was appropriate for RT planning. | Low |
| Liang et al. (2019) [33] | Patient Selection | High | Very small, non-consecutive sample (11 patients) with unclear inclusion/exclusion criteria; retrospective and convenience sampling; mismatch between title (MRI) and reported use of CT images indicates unclear population definition and spectrum bias. | High |
| Liang et al. (2019) [33] | Index Test (Deep Learning Segmentation Method) | High | The deep learning algorithm is insufficiently specified (architecture, training/testing split, hyperparameters unclear); performance metrics are non-standard for segmentation (classification-style accuracy up to 98–99%) with no Dice, HD, or spatial overlap metrics reported. | High |
| Liang et al. (2019) [33] | Reference Standard | High | No clear reference standard described for ground-truth segmentation; manual annotations, number of annotators, expertise, and blinding are not reported, making validity of “accuracy” measures uncertain. | High |
| Liang et al. (2019) [33] | Flow and Timing | High | Inconsistent reporting of imaging modality (MRI vs CT), unclear flow of cases, and lack of transparency on how images were selected, processed, and evaluated; no external validation or independent test set. | High |
| Liu et al. (2025) [34] | Patient Selection | High | Retrospective, single-center study including 165 patients from one institution; no statement on consecutive or random sampling; exclusion of patients without PET/MRI and reliance on availability of complete imaging introduces potential selection and spectrum bias. | Moderate |
| Liu et al. (2025) [34] | Index Test (PEU-Net Deep Learning Model) | Low | The index test architecture, training strategy, and evaluation metrics (DSC, HD95) were clearly pre-specified; no data-driven threshold adjustment or outcome-informed tuning was performed on the test set. | Low |
| Liu et al. (2025) [34] | Reference Standard (Manual Tumor Delineation) | High | Ground-truth contours were delineated by a single radiation oncologist (>10 years’ experience); absence of multiple annotators, inter-observer variability analysis, or consensus contouring increases risk of reference standard bias. | Low |
| Liu et al. (2025) [34] | Flow and Timing | Low | All patients underwent the same PET/MRI protocol and reference standard; consistent tra | No information |
| Ni et al. (2024) [35] | Patient Selection | Moderate | Retrospective, single-institution dataset; patients were included based on availability of MR-guided brachytherapy scans and specific applicator–scanner combinations. Although the cohort is relatively large (136 patients, 300 scans), there is no statement of consecutive or random sampling, and some scans (e.g., bladder invasion cases) were excluded from specific analyses. | Low |
| Ni et al. (2024) [35] | Index Test (DL OAR Segmentation with Transfer Learning) | Low | The nnU-Net–based model and transfer-learning strategy were clearly predefined, applied consistently, and evaluated using standard segmentation metrics (vDSC, HD95, sDSC, APL) without outcome-driven threshold optimization. | Low |
| Ni et al. (2024) [35] | Reference Standard (Manual OAR Delineation) | Moderate | Clinical contours were used as ground truth and reviewed/approved by radiation oncologists, but inter-observer variability was not assessed and blinding between contouring and model output was not reported. | Low |
| Ni et al. (2024) [35] | Flow and Timing | Low | All included scans underwent the same reference standard and index test, with patient-level data splits and consistent timing between imaging and contouring; exclusions were transparently reported for specific analyses. | Low |
| Ni et al. (2025) [36] | Patient Selection | Moderate | Retrospective, single-institution study including 56 patients (170 MR scans) with an additional internal test cohort; no explicit statement of consecutive or random sampling, which may introduce selection bias, though inclusion criteria and dataset splits were clearly described. | Low |
| Ni et al. (2025) [36] | Index Test (DL OAR Segmentation with DP Loss & wDSC) | Low | The 3D U-Net architecture, training strategy, loss functions, and evaluation metrics were predefined and consistently applied; no post-hoc threshold tuning based on test outcomes was reported. | Low |
| Ni et al. (2025) [36] | Reference Standard (Clinical OAR Contours) | Moderate | Clinical contours reviewed by experienced radiation oncologists were used, but contouring and clinical acceptability testing involved limited observers (including one fellow for acceptability/revision), and inter-observer variability was not formally quantified. | Low |
| Ni et al. (2025) [36] | Flow and Timing | Low | All patients/scans received the same reference standard and index test; patient-level data splitting avoided leakage, and an independent internal test set was used; timing reflects routine clinical practice. | Low |
| Rigaud et al., 2021 [37] | 1. Patient Selection | Low | Large retrospective multicenter dataset (408 CT scans from 247 patients across two institutions and one public challenge dataset). Clear data split into training, validation, internal test, and external test sets, with no patient overlap between cohorts. Limited exclusions were reported and justified (e.g., extreme BMI cases). | Low |
| Rigaud et al., 2021 [37] | 2. Index Test (DeepLabV3+ 2D and 3D U-Net auto-segmentation models) | Moderate | Model architectures and inference thresholds were prespecified; however, model optimization and hyperparameter tuning were performed on the same training/validation data. Although a fully independent external test set was used, the models were trained predominantly on data from one center, introducing potential center-specific bias. | Low |
| Rigaud et al., 2021 [37] | 3. Reference Standard (Manual contours) | Moderate | Manual contours were generated by experienced clinicians and reviewed by radiation oncologists and radiologists; however, no formal multi-observer consensus reference standard was used for all structures. Interobserver variability was assessed only for selected targets (CTVT) on the external test set. | Low |
| Rigaud et al., 2021 [37] | 4. Flow and Timing | Low | All CT scans received the same index tests and reference standard. Clear reporting of data flow, exclusions, and separation of internal and external test cohorts. Imaging and contouring timing reflects routine clinical practice. | Low |
| Rodríguez Outeiral et al., 2023 [43] | 1. Patient Selection | Moderate | Retrospective, single-center cohort (195 patients; 524 brachytherapy fractions). Although inclusion criteria were clearly defined and stratified splits were used, consecutive enrollment is not explicitly stated and early/late FIGO stages were under-represented. | Low |
| Rodríguez Outeiral et al., 2023 [43] | 2. Index Test (nnU-Net automatic GTV segmentation) | Moderate | Model training, validation, and testing were performed within the same institutional dataset. Although patient-level data splitting was applied, no external or multicenter validation cohort was included, raising potential overfitting/optimism bias. | Low |
| Rodríguez Outeiral et al., 2023 [43] | 3. Reference Standard (Manual GTV contours) | Moderate | Ground-truth GTVs were the clinical contours drawn by radiation oncologists for treatment planning. No multi-observer consensus or inter-observer variability analysis was performed, and clinical contours may contain geometric inaccuracies acknowledged by the authors. | Low |
| Rodríguez Outeiral et al., 2023 [43] | 4. Flow and Timing | Low | All patients received the same index test and reference standard. Clear patient-level separation into training, validation, and test sets; no partial verification or unexplained exclusions. Timing reflects routine brachytherapy workflow. | Low |
| Shi et al., 2025 [38] | 1. Patient Selection | Moderate | Retrospective single-center cohort of 222 patients. Consecutive enrollment is not explicitly reported, and only patients with pathologically confirmed cervical cancer were included, which may introduce spectrum bias. | Low |
| Shi et al., 2025 [38] | 2. Index Test (TransUNet deep learning model) | Moderate | Model development and evaluation were performed on the same institutional dataset. Although multiple planes (axial, sagittal, coronal) were evaluated, no external or independent multicenter validation cohort was used, increasing risk of overfitting. | Moderate |
| Shi et al., 2025 [38] | 3. Reference Standard (Manual lesion segmentation) | Moderate | Ground-truth segmentations are implied to be expert-annotated, but the number of annotators, consensus strategy, and inter-observer variability analysis are not reported, introducing potential subjectivity bias. | Low |
| Shi et al., 2025 [38] | 4. Flow and Timing | Low | All images underwent the same index test and reference standard. Clear reporting of dataset size and evaluation metrics, with no partial verification or unexplained exclusions. | Low |
| Xia et al., 2025 [39] | 1. Patient Selection | Low | Retrospective multicenter cohort (125 patients from two hospitals). Clear inclusion/exclusion criteria were defined, with histological confirmation and no prior treatment. Separate training, internal testing, and external cross-center testing cohorts reduce selection and spectrum bias. | Low |
| Xia et al., 2025 [39] | 2. Index Test (DL-based detection + segmentation pipeline) | Moderate | The two-stage pipeline (ConvNeXt detection + DoubleU-Net segmentation) was extensively optimized on the primary center’s data, with multiple architectures and pretrained variants explored, introducing potential model-selection bias. However, an independent external center test without fine-tuning mitigates this risk. | Low |
| Xia et al., 2025 [39] | 3. Reference Standard (Manual tumor delineation) | Low | Tumor ROIs were first delineated by a radiologist and then reviewed and corrected by a senior attending radiologist, both experienced in gynecologic imaging. This two-level expert annotation reduces reference standard bias. | Low |
| Xia et al., 2025 [39] | 4. Flow and Timing | Low | All patients received the same reference standard and index tests. Clear reporting of data flow, slicing strategy, and separation of training/validation/testing cohorts. No partial verification or unexplained exclusions. | Low |
| Xue et al., 2024 [40] | 1. Patient Selection | Moderate | Retrospective cohort of 135 patients from two hospitals. Although multicenter, the number of patients in the independent test set is small (n = 16), and consecutive inclusion is not explicitly stated, introducing potential selection bias. | Low |
| Xue et al., 2024 [40] | 2. Index Test (3D Prompt-ResUNet auto-segmentation) | Moderate | Model development involved comparison of multiple architectures (Prompt-ResUNet, nnUNet, Med3D SAM) and optimization within the same dataset, raising potential model-selection bias. No fully independent external multicenter validation cohort beyond the two hospitals. | Low |
| Xue et al., 2024 [40] | 3. Reference Standard (Expert contours) | Low | Ground truth based on expert radiation oncologist contours with interobserver comparison, 4-grade expert scoring, and a double-blinded Turing test, reducing subjectivity and strengthening the reference standard. | Low |
| Xue et al., 2024 [40] | 4. Flow and Timing | Low | All patients received the same index tests and reference standard. Clear separation into training, validation, and testing sets with no partial verification or unexplained exclusions. | Low |
| Yoganathan et al., 2022 [41] | 1. Patient Selection | Moderate | Retrospective, single-center cohort of 39 patients (71 MRI datasets). Consecutive inclusion is not explicitly stated, and multiple fractions per patient were included, which may introduce selection and dependency bias. | Low |
| Yoganathan et al., 2022 [41] | 2. Index Test (DL-based 2D / 2.5D segmentation models) | Moderate | Model development, hyperparameter tuning, and evaluation were all performed on the same institutional dataset. Although a train/validation/test split was used, no external or multicenter validation was conducted, raising risk of overfitting and optimism bias. | Low |
| Yoganathan et al., 2022 [41] | 3. Reference Standard (Manual contours) | Moderate | Ground-truth contours were delineated by an experienced radiation oncologist following GEC-ESTRO guidelines. However, no multi-observer consensus or inter-observer variability analysis was performed, and targets (especially IR-CTV) lack a true gold standard. | Low |
| Yoganathan et al., 2022 [41] | 4. Flow and Timing | Low | All MRI datasets received the same index test and reference standard. Clear data flow with defined training, validation, and test sets; no partial verification or unexplained exclusions. Timing reflects routine clinical practice. | Low |
| Zhu et al., 2024 [42] | 1. Patient Selection | Moderate | Retrospective, single-center cohort (406 patients, 2009–2016). Although inclusion/exclusion criteria were clearly defined and a large independent test set was used (n=82), consecutive enrollment was not explicitly stated, and long recruitment period may introduce spectrum bias. | Low |
| Zhu et al., 2024 [42] | 2. Index Test (CNN–Transformer segmentation model) | Moderate | Multiple architectures (U-Net, Attention U-Net, V-Net, CoTr) were compared and optimized within the same dataset. Although a predefined independent test cohort was used, no external multicenter validation was performed, raising potential model-selection and overfitting bias. | Low |
| Zhu et al., 2024 [42] | 3. Reference Standard (Manual tumor segmentation) | Low | Tumors were manually delineated by two experienced radiologists, with confirmation by a senior gynecologic oncologist. Inter-observer variability was assessed on a subset (30 patients), reducing reference standard bias. | Low |
| Zhu et al., 2024 [42] | 4. Flow and Timing | Low | All patients received the same index test and reference standard. Clear separation into training, validation, and independent test cohorts; no partial verification or unexplained exclusions reported. | Low |

**Supplementary Table S4. PICO framework and database-specific literature search strategy.**

| **PICO Element** | **Description** | **Search Terms** |
| --- | --- | --- |
| Population | Adult women with cervical cancer or related neoplasms | "cervical cancer" OR "cervix cancer" OR "cervical carcinoma" OR "cervical neoplasms"[MeSH] OR "cervix carcinoma" OR "cervix neoplasm" OR "cervical tumour" OR "cervical neoplasm*" |
| Intervention | ML/DL algorithms for MRI-based segmentation | ("deep learning" OR "machine learning" OR "artificial intelligence" OR "neural network*" OR CNN OR "U-Net" OR transformer* OR GAN OR FCN) AND (MRI OR "magnetic resonance imaging" OR "MR imaging") AND (segmentation OR "image segmentation" OR "tumor segmentation" OR "automatic segmentation" OR "lesion segmentation") |
| Comparator | Manual segmentation or traditional methods (optional) | "diagnostic performance" OR "diagnostic accuracy" OR sensitivity OR specificity OR AUC OR ROC OR "predictive value" |
| Outcome | Segmentation accuracy and performance metrics | Dice OR "Dice similarity coefficient" OR Jaccard OR IoU OR "Hausdorff Distance" OR "Average Surface Distance" OR precision OR recall OR F1-score |

**Supplementary Table S5. Reasons for exclusion of full-text articles during eligibility assessment.**

| **Reason for exclusion** | **Number** |
| --- | --- |
| Non-relevant imaging modality or absence of MRI-derived segmentation information | 42 |
| No quantitative segmentation metrics | 31 |
| Non-cervical cancer population | 18 |
| Review/meta-analysis/editorial articles | 7 |
| Total excluded after full-text review | 98 |

**Supplementary Table S6. Comparative summary of AI architectures used for cervical cancer segmentation.**

| **Architecture** | **Representative models** | **Advantages** | **Limitations** | **Clinical applications** |
| --- | --- | --- | --- | --- |
| CNN-based models | 3D CNN, Mask R-CNN | Feature extraction | Limited long-range context | Tumor segmentation |
| U-Net variants | U-Net, nnU-Net | Promising segmentation performance | Data dependency | MRI radiotherapy planning |
| Transformer models | TransUNet, CoTr | Global feature modeling | Computational demand | Complex segmentation |
| Hybrid models | CNN-transformer | Combines local/global features | Higher complexity | Clinical translation |

**Supplementary Table S7. Clinical readiness assessment of AI segmentation studies.**

| **Domain** | **Current status** |
| --- | --- |
| External validation | Limited |
| Multicenter testing | Rare |
| Regulatory approval | Not established |
| Explainability | Limited |
| Workflow integration | Early stage |
| Prospective trials | Needed |
